# Non-Toxic Increases in Nitrogen Availability Can Improve the Ability of the Soil Lichen *Cladonia rangiferina* to Cope with Environmental Changes

**DOI:** 10.3390/jof8040333

**Published:** 2022-03-23

**Authors:** Lourdes Morillas, Javier Roales, Cristina Cruz, Silvana Munzi

**Affiliations:** 1 Centre for Ecology, Evolution and Environmental Changes, Faculdade de Ciências, Universidade de Lisboa, Campo Grande, Bloco C2, 1749-016 Lisbon, Portugal; javier.roales@gmail.com (J.R.); ccruz@fc.ul.pt (C.C.); ssmunzi@fc.ul.pt (S.M.); 2 Departamento de Sistemas Físicos, Químicos y Naturales, Universidad Pablo de Olavide, Ctra. Utrera Km 1, 41013 Seville, Spain; 3 Centro Interuniversitário de História das Ciências e da Tecnologia Faculdade de Ciências, Universidade de Lisboa, Campo Grande, 1749-016 Lisbon, Portugal

**Keywords:** soil lichens, soil biocrust, global change, climate change, biomonitoring, synergetic effects, reduced watering, aridity, drylands, Mediterranean ecosystems

## Abstract

Climate change and atmospheric nitrogen (N) deposition on drylands are greatly threatening these especially vulnerable areas. Soil biocrust-forming lichens in drylands can provide early indicators of these disturbances and play a pivotal role, as they contribute to key ecosystem services. In this study, we explored the effects of different long-term water availability regimes simulating climate changes and their interaction with N addition on the physiological response of the soil lichen *Cladonia rangiferina*. Three sets of this lichen were subjected to control, reduced watering, and reduced watering and N addition (40 kg NH_4_NO_3_ ha^−1^ year^−1^) treatments for 16 months. Finally, all samples were subjected to daily hydration cycles with N-enriched water at two levels (40 and 80 kg NH_4_NO_3_ ha^−1^ year^−1^) for 23 days. We found that reduced watering significantly decreased the vitality of this lichen, whereas N addition unexpectedly helped lichens subjected to reduced watering to cope with stress produced by high temperatures. We also found that long-term exposure to N addition contributed to the acclimation to higher N availability. Overall, our data suggest that the interactions between reduced watering and increased N supply and temperature have an important potential to reduce the physiological performance of this soil lichen.

## 1. Introduction

Drylands (arid, semiarid, and dry subhumid regions receiving infrequent precipitations) cover almost 45% of the terrestrial surface, comprising Earth’s largest terrestrial biome [1]. These areas are highly vulnerable to desertification and global change [2,3], and are expected to expand due to the projected climate warming [4]. Scientists foresee that these ecosystems will respond to the future climate with important shifts in community composition and biogeochemical cycles [5,6], with global implications for carbon (C) and nutrient cycling [7,8]. Despite these facts, literature related to global change is dominated by studies performed in other ecosystems [1], leaving important questions on how key ecosystem processes in drylands will respond to global change unresolved.

Nitrogen (N) and water are the main factors limiting primary production in dryland ecosystems [9], rendering alterations to their availability highly likely to cause community-level impacts. The current context of climate change is projected to cause relevant changes in rainfall dynamics in drylands, increasing desertification and droughts [10]. This scenario urges us to understand how such alterations in highly vulnerable areas will translate into disturbed ecosystem functioning. An even more pressing issue is to reveal how interactions between these factors and other drivers of global change such as atmospheric pollutants might affect responses. Interactions can trigger complex and uncertain responses [11,12], affecting the ecosystem dynamics [13]. The N deposition in both oxidized (NO_x_) and reduced (NH_3_) forms is increasing globally due to human-induced activities [14]. In dryland ecosystems located in the Mediterranean Basin, N inputs are expected to increase from the 7 kg N ha^−1^ year^−1^ of the mid-1990s to 12 kg N ha^−1^ year^−1^ in 2050 [15]. The ability to disentangle the linkages between N deposition rates, climate change and ecosystem effects to pivotal dryland communities would substantially advance our capacity to assess and predict biotic responses to global change.

Drylands throughout the world feature sparse vegetation cover surrounded by a matrix devoid of vascular plants [16]. This matrix is frequently colonized by biocrust communities dominated by cyanobacteria, mosses and lichens, that represent a pivotal biotic component of these areas providing numerous key ecosystem services [17,18]. Lichens, which constitute one of the main components of these soils’ biocrusts, are vulnerable to atmospheric changes, which makes them highly efficient early indicators of both N deposition [19] and changes in climate [20].

As poikilohydric organisms, lichens tolerate rapidly alternate periods of deep desiccation with different levels of hydration, being able to reactivate their metabolic activity in response to small amounts of water [21]. The absence of rain events during the summer months is a typical feature of drylands. However, that does not imply that lichens are inactive during those long periods. Lichens which have trebouxioid algae as photobionts, as is the case of *Cladonia rangiferina,* can recover with not only rain or dew, but also with a high degree of air humidity [22,23]. Lichens feature a circadian cycle of metabolic activity linked to air humidity levels [24,25]. This mechanism allows them to become active after sunset, responding to decreased temperatures, and remain active up to sunrise, when they dehydrate progressively because of the increased temperature and decreased air (and thallus) humidity. This circadian hydration–dehydration cycle may be affected by the increased aridity projected for these areas in such a way that increased temperature and decreased air humidity will prevent lichens from being activated by night on daily basis. Thus, expected changes in rainfall and temperature can strongly impact the functioning of soil lichens forming the biocrust [26] and the critical ecosystem processes affected by them [27,28].

Nitrogen is an essential nutrient for lichens, playing a crucial role for many processes for both the photobiont and the mycobiont. However, N can be a stress factor if supplied in excess [29,30] and it has been related to shifts of lichen communities [31]. Given the significance of these organisms and the processes they help control, there is a growing body of literature on how lichens will respond to the expected exacerbated N deposition [32,33,34]. However, it is largely unknown how the effects of N deposition will interact with those of climate change when impacting soil lichens. Thus, the ability to assess the synergistic effects of these environmental changes through lichen-forming biocrusts would significantly increase our understanding of dryland resilience to global change.

Our goals were to (i) explore the effects of different long-term water availability regimes simulating climate changes and their interaction with N addition (40 kg h^−1^year^−1^ NH_4_NO_3_) on the physiological response of the soil biocrust-forming lichen *Cladonia rangiferina* and (ii) assess if a prior long period of exposure to different stresses determines the ability of these lichens to deal with increased N addition (40 and 80 kg h^−1^year^−1^ NH_4_NO_3_). We hypothesized that the synergistic effect of increased aridity and N addition would affect the physiological performance of *Cladonia*. Therefore, we expected (i) a lower vitality in N-treated samples exposed to reduced watering than in those exposed to one stressor only. We also expected that (ii) the long-term exposure to N addition would have contributed to the acclimation to higher N availability through activation of response mechanisms over the period. To address these hypotheses, we performed a manipulative microcosm experiment over 16 months in which the effect of reduced watering and the interaction between reduced watering and N deposition on the soil lichen *Cladonia rangiferina* were tested. We used chlorophyll *a* fluorescence as a sensitive but non-destructive approach to show how photosynthetic organisms respond to environmental stressors [35]. Disentangling the effects of multiple global change drivers on these sensitive elements of ecosystems is paramount to improving the use of lichens as bioindicators for protecting vulnerable biomes and projecting the potential consequences on ecosystem functioning.

## 2. Materials and Methods

### 2.1. Lichen Sampling

In November 2019, 50 samples of *Cladonia rangiferina* were collected from a Mediterranean maquis (see detailed description in Dias et al. [36]) with a well-developed biocrust community located in Serra da Arrábida, in the Arrábida Natural Park, south of Lisbon, Portugal (38°29′24.2″N, 9°01′58.0″W, Figure S1). Background N deposition at the site is 5.2 kg ha^−1^ year^−1^ (2.9 kg NO_x_ + 2.3 kg NH_y_) [37]. Lichens were detached from the substrate and transported to the laboratory where they were carefully cleaned to remove any impurities. All lichens were stored in trays in a well-lit area at room temperature in and were fully rehydrated before performing any measurements.

### 2.2. Incubation

The incubation was performed indoors in a well aerated location and absent from heat or cold sources, with temperature oscillating moderately according to outdoor weather. Lichen samples were contained in 50 custom-built wire mesh cages (Figure S2), each with approximately 4.5 g of lichens. Samples were installed in a vertical structure hanging from a north-facing window, ensuring that no direct sunlight reached them. Fans placed above the samples facilitated drying after treatment. To prevent vertical contamination, treatments with higher N concentrations and/or watering frequencies were placed at the bottom of the structure. The position of the samples was changed every few days to ensure homogeneous conditions.

### 2.3. Treatments

**Figure 1:**
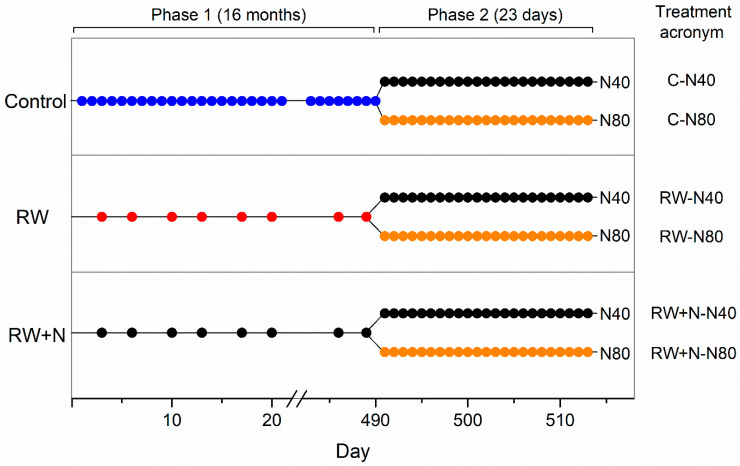
Graphical representation of the experimental design (see text for details). Dots indicate the frequency of daily artificial waterings. Control: samples subjected to one daily artificial watering cycle (blue dots). RW: samples watered twice per week (red dots). RW+N: samples watered with N-enriched water twice per week (black dots). During Phase 2, -N40 (black dots) and -N80 (orange dots) indicate the daily hydration of the samples with N-enriched water at two levels: 40 and 80 kg N h^−1^year^−1^.

As lichens have a slow growth rate, our experimental design accounted for a long-term treatment to maximize the likelihood of lichen response. The experiment consisted of two consecutive phases (Figure 1). During the first phase, which lasted 16 months, we applied three different treatments, each to a subgroup of samples: control, reduced watering (RW), and reduced watering and N deposition (RW+N). Control samples were subjected to one daily artificial watering cycle, samples testing the effect of reduced watering were watered twice per week and samples accounting for the interaction between reduced watering and N deposition were watered with N-enriched water twice per week (Figure 1). Treatments in this phase involved the immersion of the samples in mineral water, in the case of control and RW samples, or in N-enriched water (equivalent to a deposition of 40 kg N h^−1^year^−1^, N40), in the case of RW+N samples, for 30 s. Following this long-term treatment, in the second phase of our experiment, all samples were subjected to daily hydration cycles with N-enriched water at two levels: 40 and 80 kg N h^−1^year^−1^ (N40 and N80, hereafter) for 23 days (Figure 1). As fluorescence was monitored on a daily basis during this phase, it lasted long enough for lichens to respond to our short-term treatment, and the experiment ended when we found differences between treatments. Treatments consisted of a 30 s immersion of the samples in either a 17.15 mg/L or a 34.3 mg/L NH_4_NO_3_ solution, corresponding to N40 and N80. Mineral water with low mineral content was used to avoid osmotic shock on lichen tissues. The chosen N doses in this experiment were lower than the N deposition reported for other areas in Mediterranean-type ecosystems (145 kg N ha^−1^ year^−1^ [38,39]), but high enough to simulate the future projections for N deposition in this type of habitat. The concentration of N-enriched water was computed using average yearly precipitation to calculate final solution concentrations. All watering treatments were applied at around 11 AM CET so that photosynthetic processes were active during daylight. The number of replicates varied among treatments and phases, but in all cases a minimum of 5 replicates was used.

### 2.4. Chlorophyll a Fluorescence

A Handy PEA plant efficiency analyzer (Hansatech Instruments LTD, Pentney, UK) was used to quantify treatment effects through the determination of the Fv/Fm ratio, the most frequently used chlorophyll *a* fluorescence parameter in ecological research (e.g., Raggio et al. [40]; Morillas et al. [32]). The Fv/Fm ratio is used as an indicator of the photobiont vitality and a provider of an early warning of physiological stress [41]. Ten minutes after each watering treatment, once the samples were fully rehydrated, lichens were dark-adapted at room temperature for 15 min to maximize oxidation of the primary quinone electron acceptor of PSII. Then, chlorophyll *a* fluorescence was measured.

### 2.5. Statistical Analyses

Data were checked for conformity with repeated measures analysis of variance (ANOVA) assumptions using Shapiro–Wilk normality test and Mauchly’s test of sphericity. Normality tests of the residuals by time point indicated that they followed approximately a normal distribution. When sphericity could not be ensured, a Greenhouse–Geisser correction was used. Differences in Fv/Fm values among samples subjected to different treatments were evaluated via repeated measures procedure following a 2-way mixed ANOVA design with 1 within-subjects factor and 1 between-groups factor in IBM SPSS Statistics 23.0 (SPSS Inc., Chicago, IL, USA). Pairwise comparisons were performed by comparing main effects through post hoc tests using the Bonferroni correction. To investigate interactions, data were divided into subsets according to the treatments and then were subjected to repeated-measures analyses.

## 3. Results

### 3.1. Effects of Different Long-Term Water Regimes and Their Interaction with N Pollution

**Figure 2:**
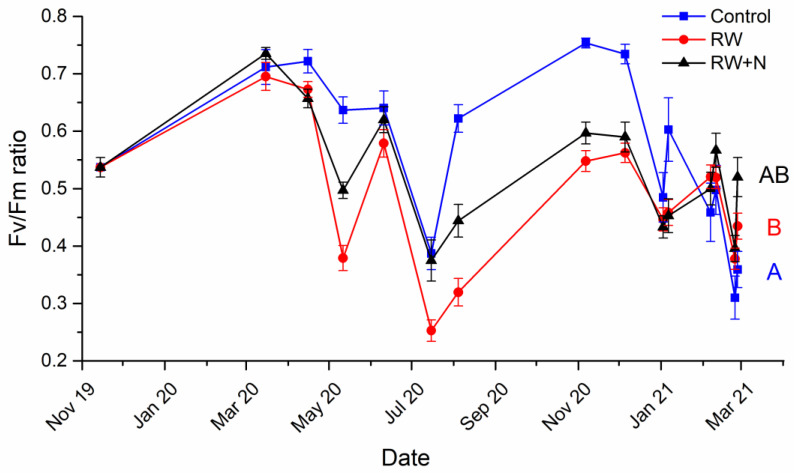
Temporal evolution of Fv/Fm ratio during phase 1 of the experiment for Control (watered every day, blue squares), RW (watered twice per week, red dots) and RW+N (watered twice per week with 40 kg N h^−1^year^−1^, black triangles) samples. Uppercase letters indicate significant differences among treatments. The study was conducted from November 2019 to March 2021.

Comparison between Control and RW samples assessed the effects of different long-term water regimes on *Cladonia* vitality (Figure 2). Significant effects of the reduced watering treatments and time were detected (Figure 2 and Table 1). Indeed, we found a general trend of decreased physiological performance in all samples subjected to reduced watering (both exposed and not exposed to N addition), although significant differences were only found between Control and RW (*p_Control/RW_* < 0.0001, *p_Control/RW+N_* < 0.141, Figure 2 and Table 1). There was a significant treatment x time interaction, which on further examination showed a significant effect of time for every treatment (Table 1). For all treatments, but mainly for those subjected to reduced watering, Fv/Fm values followed a seasonal trend: minimum values were reached coinciding with a heat wave in May 2020 and with summertime in July and August 2020 (Figure 2). To test the interactive effect of reduced watering and N pollution, we compared RW and RW+N (Figure 2). Our results showed that the physiological performance of lichens subjected to these treatments was very similar and greatest differences were observed when responding to environmental stress produced by increased temperatures (Figure 2). During these climatic conditions, N addition improved lichen vitality, although no significant differences between treatments were found (*p _RW/RW+N_* = 0.076, Figure 2 and Table 1). Lower values of measurements at the end of the experiment in the Control treatment indicated deterioration in the index due to time, i.e., the experimental conditions were likely affecting these samples disproportionally compared to the other treatments (RW and RW+N). Bleaching was observed in lichens responding to the Control treatment (Figure S3).

### 3.2. Effects of Prior N Exposure on the Ability of Cladonia to Deal with Increased N Availability

**Figure 3:**
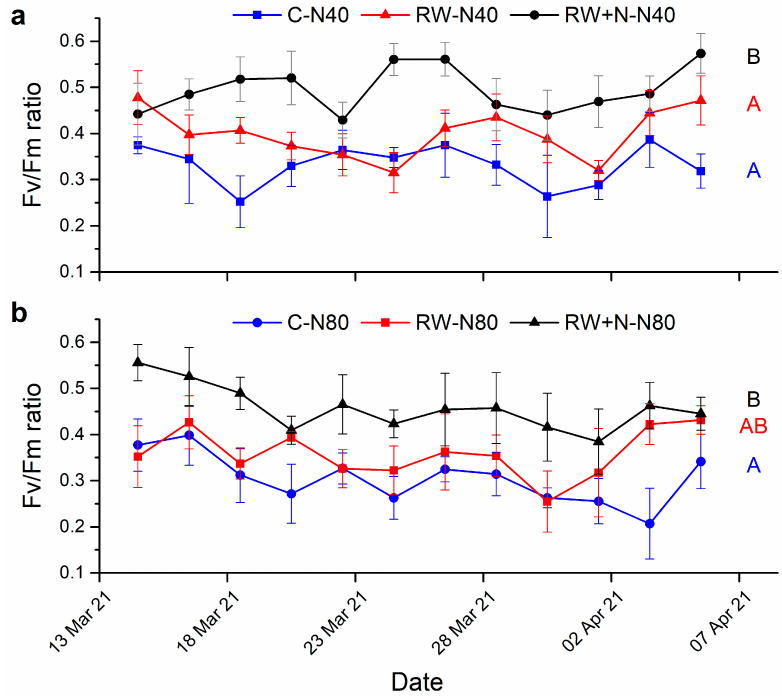
Temporal changes in Fv/Fm ratio during phase 2 of the experiment receiving 40 (**a**) and 80 (**b**) kg N h^−1^year^−1^. Control samples in phase 1 (watered every day, blue dots) are labeled as C-N40 and C-N80, respectively. RW samples in phase 1 (watered twice per week, red squares) are labeled as RW-N40 and RW-N80, respectively. RW+N samples in phase 1 (watered twice per week with 40 kg N h^−1^year^−1^, black triangles) are labeled as RW+N40 and RW+N80, respectively. Uppercase letters indicate significant differences among treatments.

Significant effects of the different N treatments were found in phase 2 of our experiment for both 40 and 80 kg N h^−1^year^−1^ (Figure 3, Table 2). Long-term N addition significantly increased Fv/Fm values for *Cladonia* samples when exposed to further N availability at both 40 (Figure 3a) and 80 (Figure 3b) kg N h^−1^year^−1^: RW+N samples showed the highest vitality values. Control samples had the lowest values while RW samples were intermediate (Figure 3a,b). Nitrogen load had no significant effect. Fv/Fm values did not follow any particular pattern over time for any treatment (Figure 3a,b).

## 4. Discussion

In increasingly arid Mediterranean areas, water is considered to be the most limiting environmental factor for primary production and microbial life [42,43], despite the direct limitations associated with the low nutrient availabilities that prevail in this ecosystem [44]. In the case of lichens, reduced water availability and high light are considered major stress components [45]. It was therefore expected that reduced water availability would be most influential, and that N addition would have a secondary, but synergistic detrimental effect. However, while our study showed a negative response of the soil lichen *Cladonia rangiferina* to reduced water availability, N addition had a positive effect (40 kg N h^−1^year^−1^), helping to ameliorate high temperature stress. Therefore, our data did not support our first hypothesis. However, our findings proved that the long-term exposure to N addition contributed to the acclimation to higher N availability, supporting our second hypothesis.

Biocrust lichens in our experiment proved to be particularly responsive to reduced watering. The sensitivity of lichens to changes in humidity is related to their poikilohydric nature. The water content of their cells is in equilibrium with the surrounding environment and their physiological activity is limited to periods of hydration. Thus, performance and growth during each hydration event are key factors that will determine their long-term survival. In poikilohydric organisms, each wetting event induces a period of net carbon loss due to respiration, reparation, and reinstatement of metabolism. When photosynthesis starts, carbon fixation exceeds respiration, which leads to net carbon gains during the wet period. When tissues desiccate, photosynthesis ceases, leading to a phase of carbon loss [46]. Therefore, in drylands, each hydration event involves a balance between energy spent during rehydration and energy gained while hydrated, which leads to an overall positive or negative carbon balance [47]. When assessing longer time scales, carbon balances from individual wetting events are tightly linked to long-term growth and survival. The timing of wetting events and the length of desiccation period are coupled, which determines the lichen carbon balance. Lichens progressively drying in early morning as a result of slow temperature rise will likely have a more positive carbon balance and survival than lichens drying suddenly as a result of high temperature. We believe that the declining Fv/Fm values reported for the control samples at the end of phase 1 in this study are due to the cumulative effect of negative carbon balance resulting from daily watering. As watering treatment of control samples was applied daily around 11:00 CET, air temperature was likely to exceed those to which lichens are exposed following the morning dew. This more rapid drying led to a negative carbon balance. In agreement, Maphangwa et al. [48] found that the highest effective photosynthetic quantum yield was measured in lichens before 10:00 h and the lowest around the solar noon. Temperatures above 20–25 °C irreversibly decreased the photosynthetic rate in lichens in arid deserts [49] and above 33 °C in a hot steam vent area of Hawaii [50]. Sixteen months of daily watering and potentially negative carbon balance affected the vitality of our samples, indicated by the lower Fv/Fm values, reducing their ability to tolerate phase 2 of our experiment. Damage from rapid desiccation in other poikilohydric organisms has been reported to involve cell membrane leakage of ions and electrolytes and reduced chloroplast and mitochondrial membrane integrity [51]. Importantly, loss of chlorophyll, inhibited growth rates [52,53] and bleaching [54,55] have also been reported as a result of rapid desiccation in mosses. Accordingly, we have also found severe bleaching in lichens responding to control versus other treatments.

The absence of rainfall in dryland ecosystems commonly forces poikilohydric organisms such as *Cladonia rangiferina* to rely on hydration (and therefore activity) with each morning dew. Those metabolically active periods are also necessary for ROS-scavenging enzymes and other anti-oxidative molecules to work [56,57], because their activity is absent at low thallus water contents [58]. The observed negative impact of reduced length of the wet, metabolically active periods on *Cladonia*’s vitality can be therefore explained by a constrained carbon gain [59] and a lack of protection from ROS and oxidative stress. The ability of soil lichens to uptake carbon was likely dramatically decreased due to reductions in their wet daytime period and net photosynthesis. Accordingly, Maphangwa et al. [48] found, in an open-top warming chamber experiment, that the interaction of climate warming with reduced precipitation increased desiccation damage and reduced the duration of photosynthetic activity. Li et al. [60] recently demonstrated that annual carbon fixation of lichen-dominated biocrust was promoted in wet years because soil lichens have a longer wet daytime period for the uptake of available carbon via photosynthesis. In fact, biocrust photosynthesis seems to be determined by the water content rather than either photosynthetically active radiation or temperature [60,61].

The lowest Fv/Fm values in RW samples were observed in July and August, suggesting that increased temperatures can exacerbate effects in samples subjected to reduced watering. Larson [62] documented that pre-treatment with increased temperature were able to produce a negative carbon balance in lichen species of the genus *Umbilicaria* and that the longer the duration of the exposure, the lower the temperature needed to affect net photosynthesis and gas exchange. The coupling of carbon deficits with high temperatures and constrained moisture was plausibly used also for explaining similar results in biocrusts dominated by mosses [63]. Warming reduced the cover, richness and evenness of lichen-dominated biocrusts in drylands [64]. Therefore, the photosynthetic activities of lichens and carbon fixation are expected to be sensitive to warming in the short term [48].

Nitrogen addition improved the vitality of soil lichens subjected to reduced watering. Although not significant, differences were found between RW and RW+N (*p* = 0.076), suggesting that N supply increased *C. rangiferina*´s vitality, particularly when facing heat stress. As for previously studied terricolous lichens [65], *C. rangiferina* lacks mechanisms to down-regulate their N uptake with increasing N load. Most lichens have evolved in N-poor ecosystems [66] and are therefore adapted to low-nutrient environments and take up high amounts of resources whenever available [67]. While we expected that our long-term N treatment would have a toxic effect on *C. rangiferina*, our findings implied that the N addition treatment was below the toxicity threshold, which could be because the treatment duration was insufficient for the N to become toxic, or the concentrations were tolerable. It is possible that the N-fertilized lichens in this experiment used the absorbed N to increase investment in its photosynthetic capacity, as observed in other fertilization studies with epiphytic lichens [68]. Accordingly, proteomic analyses proved that *Cladonia portentosa* invested in proteins linked to the energetic metabolism in response to N stress [69].

In agreement with our second hypothesis, the long-term exposure to N addition contributed to the acclimation to higher N availability along the time. The ability to cope with long-term N depositions has already been observed in other *Cladonia* species [19]. This is in line with the hypothesis that tolerance to increased N may be aligned with the capacity to maintain balanced carbon to N stoichiometry among the symbiont partners when high N rates are supplied through the investment of the extra N into more photobiont cells [70]. Increased energy production has also been observed in *Cladonia portentosa* to support changes in the mycobiont proteome in response to N stress [69]. It is interesting to note that there were no significant differences between the response of *Cladonia rangiferina* to 40 and 80 kg N ha^−1^ year^−1^ for any treatment, indicating that this lichen can acclimate to large variations in environmental N supply before reaching the toxicity threshold.

Many studies have underlined the complex interplay between climate and air pollution [71,72,73,74], the mainstream idea being, for a long time, that the lichen mainly responds to pollutants. This work indicates that increased aridity can be a major critical factor limiting lichens’ vitality, a hypothesis already explored by other authors [75,76,77].

We coupled the physiological responses observed in this experiment with four alternative future scenarios of global change accounting for the interplay of alterations in aridity, temperature, and N deposition to form qualitative projections. The four possible scenarios presented in Table 3 were elaborated according to the projections of a large number of Global Circulation Models belonging to the Coupled Model Intercomparison Project Phase Six (CMIP6) and N deposition global scale evaluations [78] for Mediterranean areas located in Europe in the next century. Most climate models project an overall increased aridity in this area that could be coupled or not with increased N deposition (Scenarios 1 and 2 in Table 3). Based on the results from this study, both scenarios would moderately decrease *C. rangiferina*'s vitality. Scenario 3, accounting for increases in all the three factors considered in this study (aridity, N deposition and temperature), would cause a more acute detrimental effect on this lichen's fitness. Even more severe consequences would be drawn from Scenario 4, as increased aridity and temperature without a raise in N supply under the toxicity threshold would cause a dramatic reduced fitness in *C. rangiferina*.

Projections of dryland organism response to global change remain under debate. Some studies suggest plant and soil food webs of these ecosystems are relatively resilient to climate changes, as they are adapted to extreme environments [79,80]. Our study, along with others, suggests that many dryland organisms are already living at the edge of their ecological tolerance and could strongly and non-linearly respond to even subtle changes in climate [81,82,83,84].

## 5. Conclusions

The work presented here suggests that (1) reduced watering was a key factor decreasing the vitality of the soil lichen *Cladonia rangiferina*; (2) N addition improved stress tolerance of high temperatures; and (3) the long-term exposure to N addition contributed to the acclimation to higher N availability. Despite conventional thought that increased N will affect (even linearly) ecophysiological responses in soil lichens, this work illustrates that the actual behavior depends on interactions with other drivers of global change. The interactions between reduced watering, increased N supply and temperature that may occur in this region have the potential to adversely affect the physiological performance of this soil lichen. *Cladonia rangiferina* and, presumably, other lichens with similar ecology can cope better with increased aridity if they benefit from increased N supply below the toxicity threshold. Decreased physiological performance of biocrust-forming organisms in dryland regions may likely alter the complex structure and multifunctionality of Mediterranean ecosystems [85]. Therefore, it is urgent for stakeholders to develop suitable countermeasures for the conservation of these areas. These data will improve our understanding of how changing factors such as climate and pollution will impact dryland soil lichens and will inform forecasts of how these populations may respond to the variety of disturbances they will face in a changing future.

## Figures and Tables

**Table 1:** Repeated measures ANOVA analyses for all treatments during phase 1 of our experiment. Control = watering every day, RW = Reduced watering (twice per week) and RW+N = Reduced watering (twice per week) and nitrogen addition. df: degrees of freedom, F: F-value, *p*: *p*-value.

Factor	df	F	*p*
Treatment	2	11.810	<0.0001
Time	7.622	39.155	<0.0001
Treatment × Time	15.245	5.578	<0.0001
Interaction Control	4.238	18.835	<0.0001
Interaction RW	6.525	27.626	<0.0001
Interaction RW+N	3.384	9.713	<0.0001

**Table 2:** Repeated measures ANOVA analyses for all treatments during phase 2 of our experiment. df: degrees of freedom, F: F-value, *p*: *p*-value.

Factor	df	F	*p*
40 kg N h^−1^year^−1^ (N-40)			
Treatment	2	12.293	0.001
Time	11	1.385	0.187
Treatment × Time	22	1.054	0.406
80 kg N h^−1^year^−1^ (N-80)			
Treatment	2	5.566	0.019
Time	11	2.309	0.013
Treatment × Time	22	0.703	0.830

**Table 3:** Projections for the vitality of *Cladonia* and other lichens with similar ecology based on four global change scenarios for the next century. Components of each scenario were gathered from the literature, and predictions for lichen vitality were made based on outputs from this study. Arrows pointing upward indicate an increasing tendency, while those pointing downward represent a decrease. More arrows indicate a greater effect.

Scenario	Aridity	Nitrogen Deposition	Temperature	Lichen Vitality
1	↑	-	-	↓
2	↑	↑	-	↓
3	↑	↑	↑	↓↓
4	↑	-	↑	↓↓↓

## Data Availability

The data presented in this study are openly available in FigShare at https://doi.org/10.6084/m9.figshare.19130792 (accessed on 21 March 2022).

## Supplementary Materials

The following supporting information can be downloaded at: https://www.mdpi.com/article/10.3390/jof8040333/s1, Figure S1: Map of the sampling site; Figure S2: Pictures of the custom-built wire mesh cages containing lichen samples; Figure S3: Pictures of the lichen samples in different stages of the experiment.
