# Supplementary material for: Non-Toxic Increases in Nitrogen Availability Can Improve the Ability of the Soil Lichen Cladonia rangiferina to Cope with Environmental Changes
Source: J Fungi (Basel). 2022 Mar 23;8(4):333. doi: 10.3390/jof8040333 (PMC9025437; doi:10.3390/jof8040333)

Non-toxic increases in nitrogen availability can improve the ability of the soil lichen *Cladonia rangiferina* to cope with environmental changes

Lourdes Morillas [1,*], Javier Roales [2,1], Cristina Cruz [1] and Silvana Munzi [3,1]

[1] Centre for Ecology, Evolution and Environmental Changes, Faculdade de Ciências, Universidade de Lisboa, Campo Grande, Bloco C2, 1749-016 Lisbon, Portugal
[2] Departamento de Sistemas Físicos, Químicos y Naturales, Universidad Pablo de Olavide, Ctra. Utrera Km 1, Seville, Spain
[3] Centro Interuniversitário de História das Ciências e da Tecnologia Faculdade de Ciências, Universidade de Lisboa, Campo Grande, 1749-016, Lisbon, Portugal

* Correspondence: lmorillas@fc.ul.pt (L.M.)

Figure S1. Maps of the sampling site. © OpenStreetMap contributors. Licensed under the Open Data Commons Open Database License (ODbL) by the OpenStreetMap Foundation (OSMF).

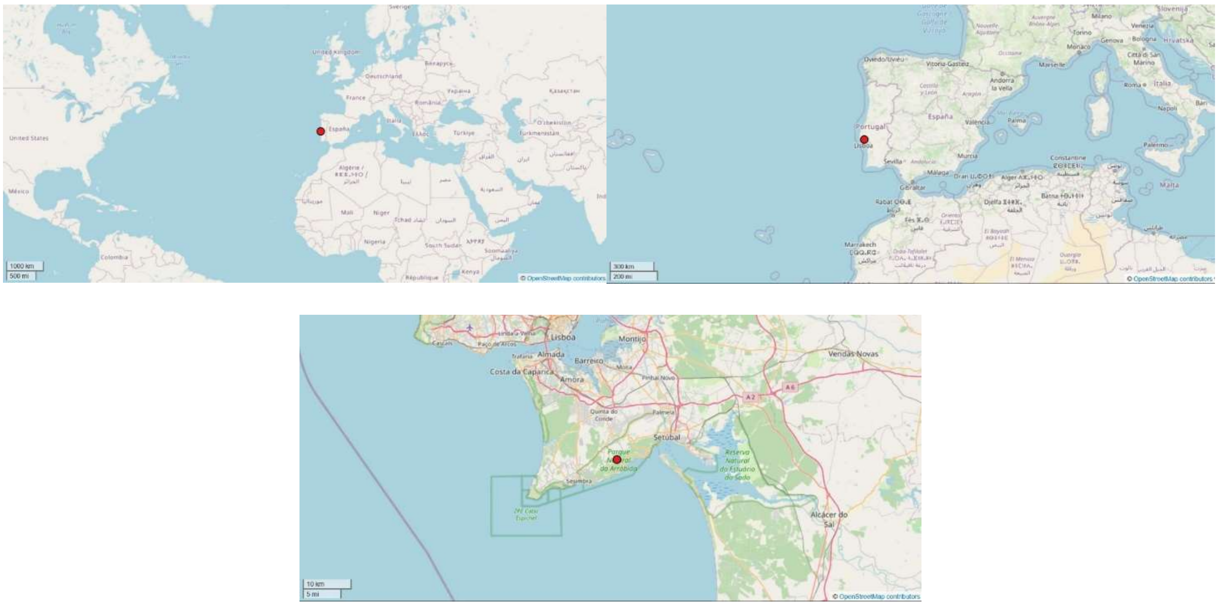

Figure S2. Pictures of the custom-built wire mesh cages containing each one approximately 4.5 g of lichens. Cages were installed in a vertical structure hanging from a window with fans on top of the samples to promote drying after watering treatments.

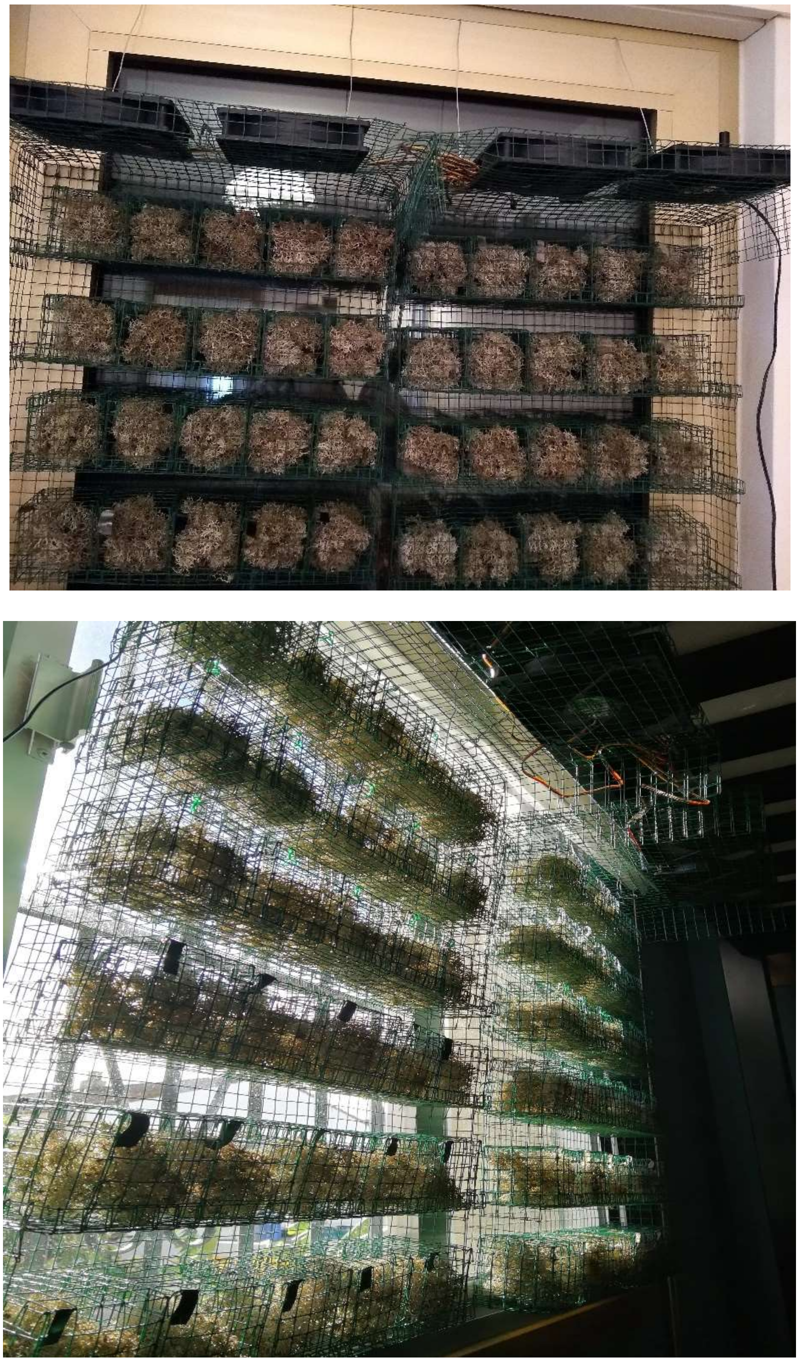

Figure S3. Pictures of (a) lichen being collected from the field, (b) lichens placed in the custom-built wire mesh cages, and (c) lichens responding to Control treatment (watered everyday) at the end of phase 1 with signs of bleaching.

a.

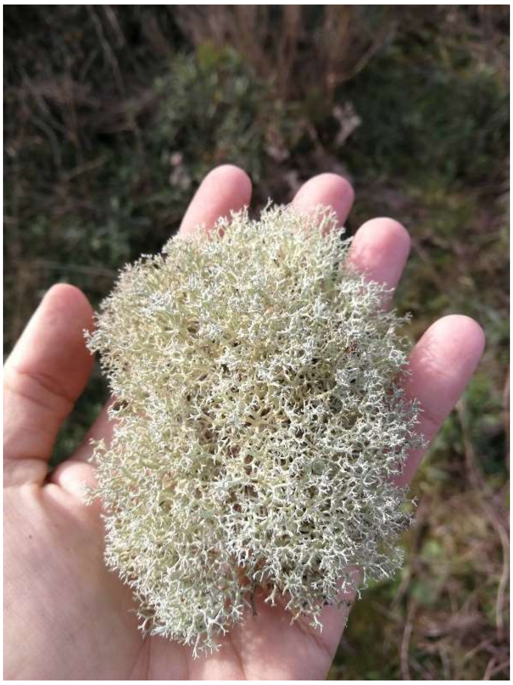

b.

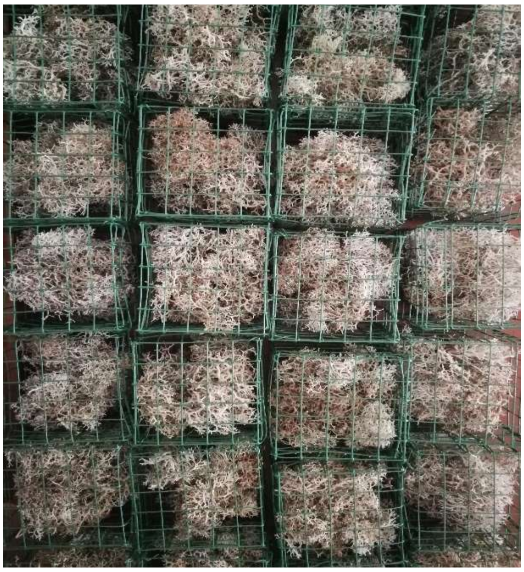

c.

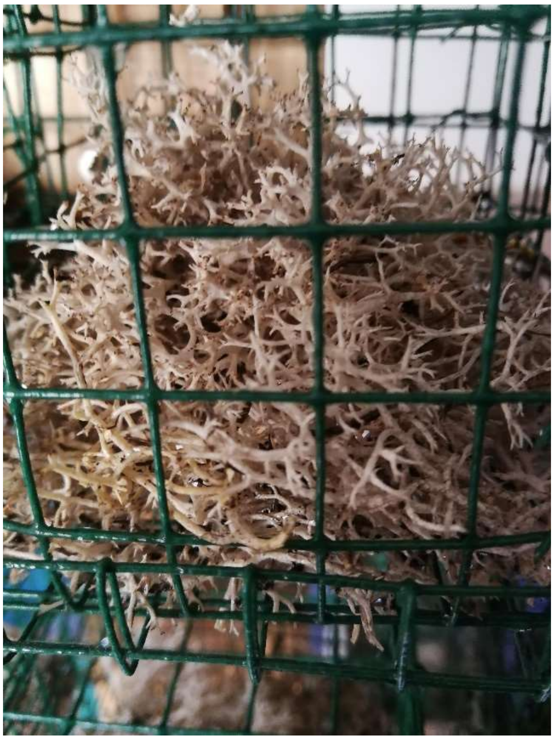

Supplement: Supplementary file 1 [file jof-08-00333-s001.zip › jof-1609753-supplementary.pdf]
